# Wildlife as sentinel of antimicrobial resistance in *Klebsiella* spp. with genomic insights into *Klebsiella pneumoniae* in Northern Italy

**DOI:** 10.3389/fmicb.2026.1716432

**Published:** 2026-04-16

**Authors:** Maria Sampieri, Lia Bardasi, Silvia Bonardi, Ilaria Menozzi, Alessandra Dodi, Erika Scaltriti, Giorgio Galletti, Elisa Massella, Cristina Bacci, Martina Rega, Mauro Conter

**Affiliations:** 1 Istituto Zooprofilattico Sperimentale della Lombardia e dell’Emilia-Romagna, Bologna, Italy; 2 Department of Veterinary Science, Unit of Food Safety, University of Parma, Parma, Italy; 3 Risk Analysis and Genomic Epidemiology Unit, Istituto Zooprofilattico Sperimentale della Lombardia e dell'Emilia-Romagna, Parma, Italy; 4 Epidemiology Unit, Istituto Zooprofilattico Sperimentale della Lombardia e dell'Emilia-Romagna, Bologna, Italy

**Keywords:** antimicrobial resistance, carbapenemases, colistin, extended-spectrum *β*-lactamase, *Klebsiella pneumoniae*, multidrug resistance, red fox, wildlife

## Abstract

**Introduction:**

Antimicrobial resistance (AMR) is a global concern, particularly worsened by the spread of multidrug-resistant (MDR) bacteria such as *Klebsiella pneumoniae*.

**Methods:**

This study investigated the presence of extended-spectrum *β*-lactamase (ESBL) and carbapenemase-producing *K. pneumoniae* in wild birds (corvids and waterfowl) and in red foxes in Northern Italy.

**Results and discussion:**

Although the overall prevalence of *K. pneumoniae* in the present study was relatively low (2.0, 95% CI: 1.0–3.7%), its isolation from multiple wild species confirms the environmental circulation of this clinically relevant AMR bacterium in ecosystems not directly exposed to antibiotic pressure. In particular, the isolation of a carbapenemase-producing strain from a fox highlights the introduction of clinically significant carbapenemase genes into wildlife reservoirs. The phenotypic AMR profiles of *K. pneumoniae* isolates revealed a high prevalence of MDR strains that were largely confirmed by the genomic analysis. *In-silico* analyses of *K. pneumoniae* sequencing data led to the identification of the most frequent resistance genes and the *in-silico* typing reveals the prevalence of the ST307 high-risk clone. Remarkably, wildlife could be considered a significant AMR sentinel, serving as carriers of MDR *K. pneumoniae* in different and even distant geographic areas. These findings underscore the importance of integrating wildlife surveillance to monitor the environmental spread of AMR within a One Health approach.

## 1 Introduction

Antimicrobial resistance (AMR) in microorganisms affecting human health is a threat worldwide (Dziekan et al., 2012). Third-generation cephalosporins (3GCs), such as cefotaxime and ceftazidime, and carbapenems, such as meropenem and imipenem, are considered critically important antimicrobials (CIA) in human medicine according to the World Health Organization (2019). In human medicine, 3GCs are used to treat severe Gram-negative and Gram-positive bacterial infections (such as meningitis, endocarditis, pneumonia, osteomyelitis, Lyme disease) (Arumugham et al., 2024). Carbapenems are used to treat infections by multidrug resistant (MDR) bacteria, as well as severe infections by extended-spectrum *β*-lactamase (ESBL) producing microorganisms (Partier and Timsit, 2020). Due to their therapeutic importance, resistance to these antimicrobials has received increased attention worldwide.

Resistance to 3GCs is mediated by different ESBL-encoding genes (*bla*_TEM,_ *bla*_SHV_, *bla*_CTX − M_ and *bla*_VEB_) and AmpC-encoding genes (*bla*_CMY,_ *bla*_FOX,_ *bla*_DHA_ and *bla*_MOX_) (Bonomo, 2017). Resistance to carbapenems is mediated by *β*-lactamases called carbapenemases, which are divided into different classes based on their structural properties. Class A includes the *bla*_KPC_, *bla*_IMI_, *bla*_GES_, and *bla*_SME_ genes encoding serine-enzymes (Bonomo, 2017), class B gathers the *bla*_IMP_, *bla*_VIM_ and *bla*_NDM_ genes encoding metallo-enzymes with the highest carbapenemase activity (Nordmann et al., 2011) and class D groups the *bla*_OXA_ genes encoding carbapenem-hydrolyzing oxacillinases (OXA) (serine-enzymes) (Naas et al., 2017). These genes are commonly located on mobile genetic elements (MGE) such as plasmids or transposons. They often coexist with additional resistance determinants active against classes of antimicrobials such as aminoglycosides, co-trimoxazole, quinolones and other antimicrobials, giving rise to MDR bacteria (Dolejska and Papagiannitsis, 2018; Rozwandowicz et al., 2018). The horizontal transfer of resistance genes among microorganisms, and particularly in the *Enterobacteriaceae* family, contributes to the spread of resistant bacterial strains, significantly interfering with antibiotic treatments (Salam et al., 2023).

Among human pathogens, *Klebsiella pneumoniae* is included in a list of bacteria whose acronym is ESKAPE (*Enterococcus faecium*, *Staphylococcus aureus*, *Klebsiella pneumoniae*, *Acinetobacter baumannii*, *Pseudomonas aeruginosa*, and *Enterobacter* spp.). Indeed, due to their resistance to antimicrobials, they are able to “escape” the biocidal action of many antibacterial agents causing healthcare associated infections with high mortality risk (Navidinia, 2016). Among antimicrobial-resistant *K. pneumoniae*, the strains producing ESBLs and carbapenemases are of particular concern because they are responsible for urinary tract, lower respiratory tract, intra-abdominal, and bloodstream infections in hospitalized patients, and are often associated with high mortality [ECDC (European Centre for Disease Prevention and Control), 2020]. Data from the ECDC and WHO indicate that in 2021, 53.3 and 26.7% of invasive *K. pneumoniae* isolates from patients in Italy were resistant to 3GCs and carbapenems, respectively (ECDC and WHO, 2023).

When clinically relevant AMR genes and antibiotic-resistant bacteria are found in wild animals (not receiving antibiotics) this should be considered as a marker of AMR pollution (Samreen et al., 2021; Quintelas et al., 2024). Wildlife can act as reservoirs for AMR zoonotic bacteria, which may be transferred to domestic animals and humans, leading to a challenging cycle of resistance (Mo et al., 2018; O’Hagan et al., 2021; Plaza-Rodríguez et al., 2021; Laborda et al., 2022). The wide ecological distribution of *K. pneumoniae* in wastewater, saltwater and freshwater environments, as well as in fresh vegetable production sites (Wyres and Holt, 2018), has led to the detection of ESBL-producing strains in various environments and animal species. Among them, wild boars, fallow deer, red deer, row deer, European baggers, red foxes, wolves and magpies (Bonardi et al., 2019; Chiaverini et al., 2022), gulls (Bonnedahl et al., 2014) and migratory birds (Raza et al., 2017) can be cited. These findings support the hypothesis that wild animals could play a central role in the spread of CIA-resistant *K. pneumoniae* strains in different habitats and areas, making more difficult to counteract AMR.

Among wild animals, birds are considered key sentinels due to their ability to cover long distances and live in different urban, suburban, and livestock environments. The variety of niches they occupy reflect different anthropic activities, including hospitals and health care facilities, thus transforming them in carriers of human, animal, and environmental microorganisms (Bonnedahl et al., 2009; Guenther et al., 2010; Radhouani et al., 2012; Gambino et al., 2021; Di Francesco et al., 2023). Among mammals, red foxes can play a significant role as AMR indicators, especially in areas characterized by high population density, whereas they live associated with human activities (Mo et al., 2018). Therefore, surveillance of AMR in wildlife is crucial, as it offers essential insights into AMR routes and guides strategies for monitoring and prevention.

The aims of this study were: (i) monitoring of ESBL- and carbapenemase-producing *K. pneumoniae* in wild animals (corvids, waterfowl and red foxes) in Emilia-Romagna region, Northern Italy; (ii) characterize ESBL- and carbapenemase-producing *K. pneumoniae* virulence and AMR traits by Whole Genome Sequencing (WGS) to evaluate the dynamics of resistance patterns.

## 2 Materials and methods

### 2.1 Sampling

In the framework of the Wildlife Monitoring Plan of Emilia-Romagna Region (Italy), 493 samples were collected from wild animals that died as a result of trauma or predation between August 2020 and February 2023, representing a convenience sampling approach. Out of 493 animals, 184 (37.2%) were red foxes (*Vulpes vulpes*), 86 (17.4%) crows (*Corvus corone cornix*), 123 (25.0%) magpies (*Pica pica*) and 100 (20.3%) water birds that belonged to different species such as herons (genus *Ardea*), swans (*Cygnus olor*), mallards (*Anas platyrhynchos*), grebes (*Podiceps cristatus*), teals (*Anas crecca*), shovelers (*Spatula clypeata*), flamingos (*Phoenicopterus roseus*), and black-winged stilt (*Himantopus himantopus*). The animals have been aggregated into three main groups according to their species and/or habitats: foxes (184 animals, 37.2%), corvids, including crows and magpies (209 animals, 42.4%) and waterfowl (100 animals, 20.3%).

**Figure 1:**
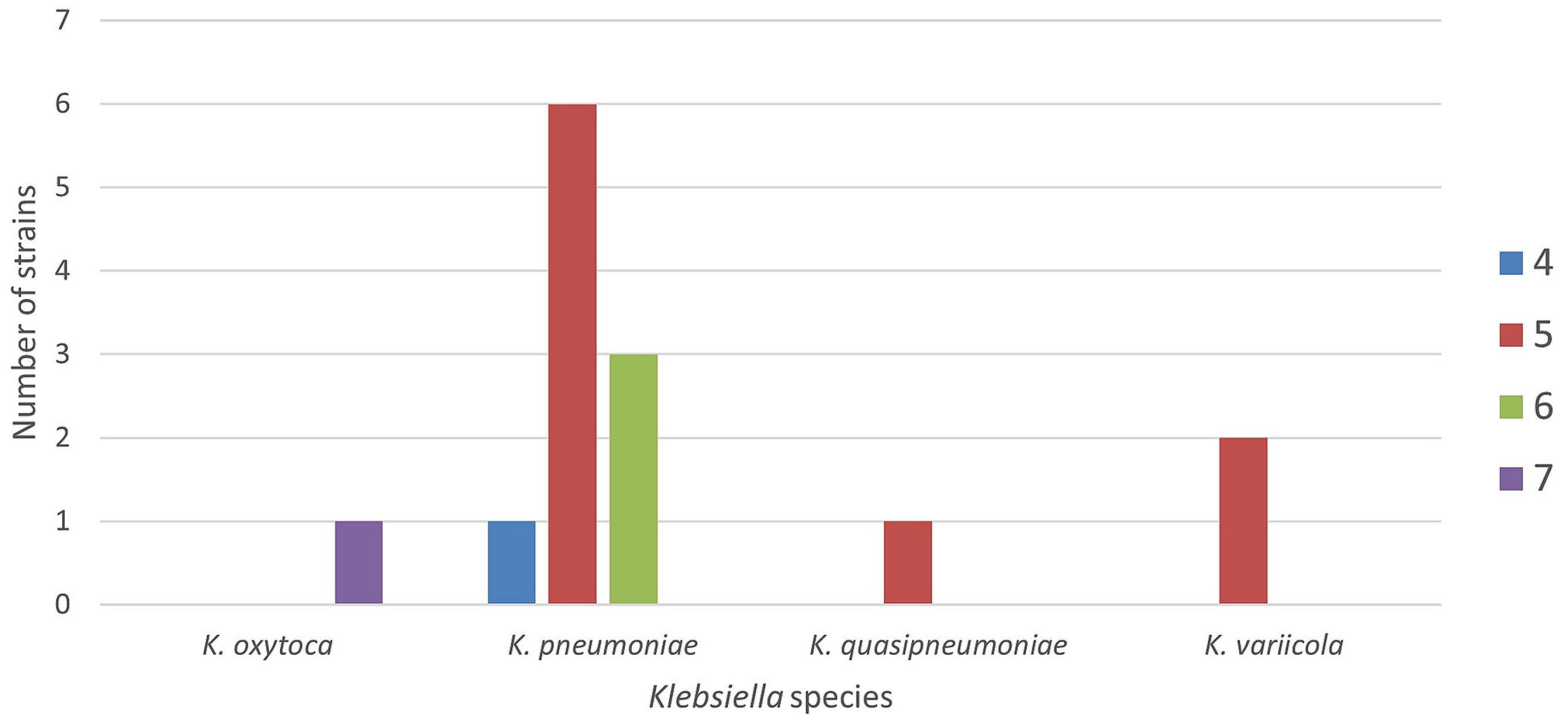
Spatial distribution of the animals sampled in the Emilia-Romagna region by municipalities and animal group.

For each animal, the location of recovery was recorded to assess the territorial coverage. The distribution of the sampled animals on the regional territory is shown in Figure 1.

### 2.2 Microbiological analysis

The fecal samples were aseptically collected from the gut of the animals and tested for *Klebsiella* spp. following the standardized and internationally recognized “Laboratory Protocol, Isolation of ESBL, AmpC and carbapenemase-producing *E. coli* from caecal samples, Version 7” (2019) provided by the DTU (Technical University of Denmark) National Food Institute. Briefly, 1 g of faeces was seeded into 9 mL of Buffered Peptone Water (BPW, Oxoid, Basingstoke, UK) and incubated at 37 ± 1 °C for 18–22 h. The enrichment broths were streaked onto selective media for cefotaxime-resistant Enterobacterales (MacConkey agar supplemented with 1 mg/L of cefotaxime) and for carbapenem-resistant bacteria (CHROMID™ CARBA SMART; bioMérieux, Marcy l’Etoile, France). All plates were incubated at 37 ± 1 °C for 18–22 h. The CHROMID™ CARBA SMART medium allows the presumptive and differential identification of *E. coli* and *K. pneumoniae* thanks to its chromogenic substrates. To obtain pure cultures, *Klebsiella* suspected colonies grown in the two media were plated onto Tryptone Soya Agar (TSA, Oxoid) and incubated at 37 ± 1 °C for 18–22 h. Species identification of single isolates was carried out by the MALDI-TOF technique (Biotyper system^®^ Bruker Daltonics).

### 2.3 Antimicrobial susceptibility test

*Klebsiella* spp. isolates were tested for sensitivity to the panel of antimicrobials recommended by the Commission Implementing Decision 2020/1729/EU (European Commission, 2020) by using Sensititre™ EUVSEC3 plates (ThermoFisher Scientific, East Grinstead, UK). The groups of tested antimicrobials were aminoglycosides (amikacin–AMK, gentamicin–GEN), penicillins (ampicillin–AMP), 3GCs (cefotaxime–CTX, ceftazidime–CAZ), carbapenems (meropenem–MEM), phenicols (chloramphenicol–CHL), quinolones (nalidixic acid–NAL), fluoroquinolones (ciprofloxacin–CIP), macrolides (azithromycin–AZM), polymixins (colistin–COL), folate pathway antagonists (sulfamethoxazole–SMX, trimethoprim–TMP), tetracyclines (tetracycline–TET) and glycylcyclines (tigecycline–TIG). Categorization of the isolates was based on the European Committee on Antimicrobial Susceptibility Testing (EUCAST) 2020 breakpoints [EUCAST (European Committee on Antimicrobial Susceptibility Testing), 2020]. *E. coli* ATCC 25922 (sensible to all antibiotic tested) was used as quality control microorganism. Multidrug-resistant (MDR) isolates were defined as those isolates resistant to at least one antibiotic molecule in ≥3 antimicrobial classes, not considering intrinsic resistance (e.g., ampicillin, to which *K. pneumoniae* isolates are known to be resistant) (Magiorakos et al., 2012; Wyres et al., 2020). The multiple antibiotic resistance (MAR) index was estimated by following the formula *a*/*b*, where *a* = the number of antibiotics to which the isolate was resistant, *b* = the number of antibiotics to which the isolate was exposed (Krumperman, 1983).

### 2.4 Whole-genome sequencing

Due to their public health relevance, only the *K. pneumoniae* isolates were submitted to WGS to characterize their genomic content. Genomic DNA was extracted by Maxwell(R) HT 96 gDNA Blood purification kit (Promega, Madison, USA) and used to prepare genomic libraries using Illumina^®^ DNA Prep (M) Tagmentation kit (Illumina, San Diego, CA). Paired-end raw reads (250 × 2 or 300 × 2 bp) were obtained using Illumina MiSeq platform (Illumina, San Diego, CA) and then checked for quality and for bacterial species identity. Reads were trimmed with Trimmomatic ver. 0.38 (Bolger et al., 2014), assembled using Unicycler ver. 0.4.8 (Wick et al., 2017) and assembly graphs were visualized through Bandage (Wick et al., 2015) to assess assembly completeness and to identified closed plasmids for each assembly.

In addition, the carbapenem-resistant strain No 94 was sequenced using long read technology through PacBio Single-Molecule Sequencing: the Nanobind CBB Kit was used for the extraction of HMW DNA and genomic library were prepared using SMRTbell prep kit 3.0. High quality *de novo* assemblies of HiFi reads were performed by using SMRT Link software.

The Multi Locus Sequence Type (ST) was *in silico* detected using Pasteur BIGSdb for *K. pneumoniae*.^1^ Starting from genome assemblies, AMR genes, a set of virulence loci, namely yersiniabactin, colibactin, aerobactin and salmochelin loci, as well as the derived virulence score, K and O loci and capsule type genes were *in-silico* detected using Kleborate v3 (Lam et al., 2021).

Mobile Element Finder and ResFinder software were used for *in silico* detection of mobile genetic element harboring resistance genes, on assemblies (Camacho et al., 2009; Bortolaia et al., 2020). PlasmidFinder 2.1 was used for plasmid detection and replicon type identification (Johansson et al., 2021).

BWA-MEM was used to evaluate if reads related to AMR plasmid, identified in strain No 94, were shared between Kp strained collected in this work.

### 2.5 Statistical analysis

To assess whether the distribution of *Klebsiella* species differed significantly across animal groups, a Chi-square test was performed.

All the statistical analyses were performed in SPSS 29 (IBM Corp. Released 2020, IBM SPSS Statistics for Windows, Version 29.0. Armonk, NY, USA: IBM Corp).

## 3 Results

### 3.1 Bacterial detection and identification of *Klebsiella* spp.

**Figure 2:**
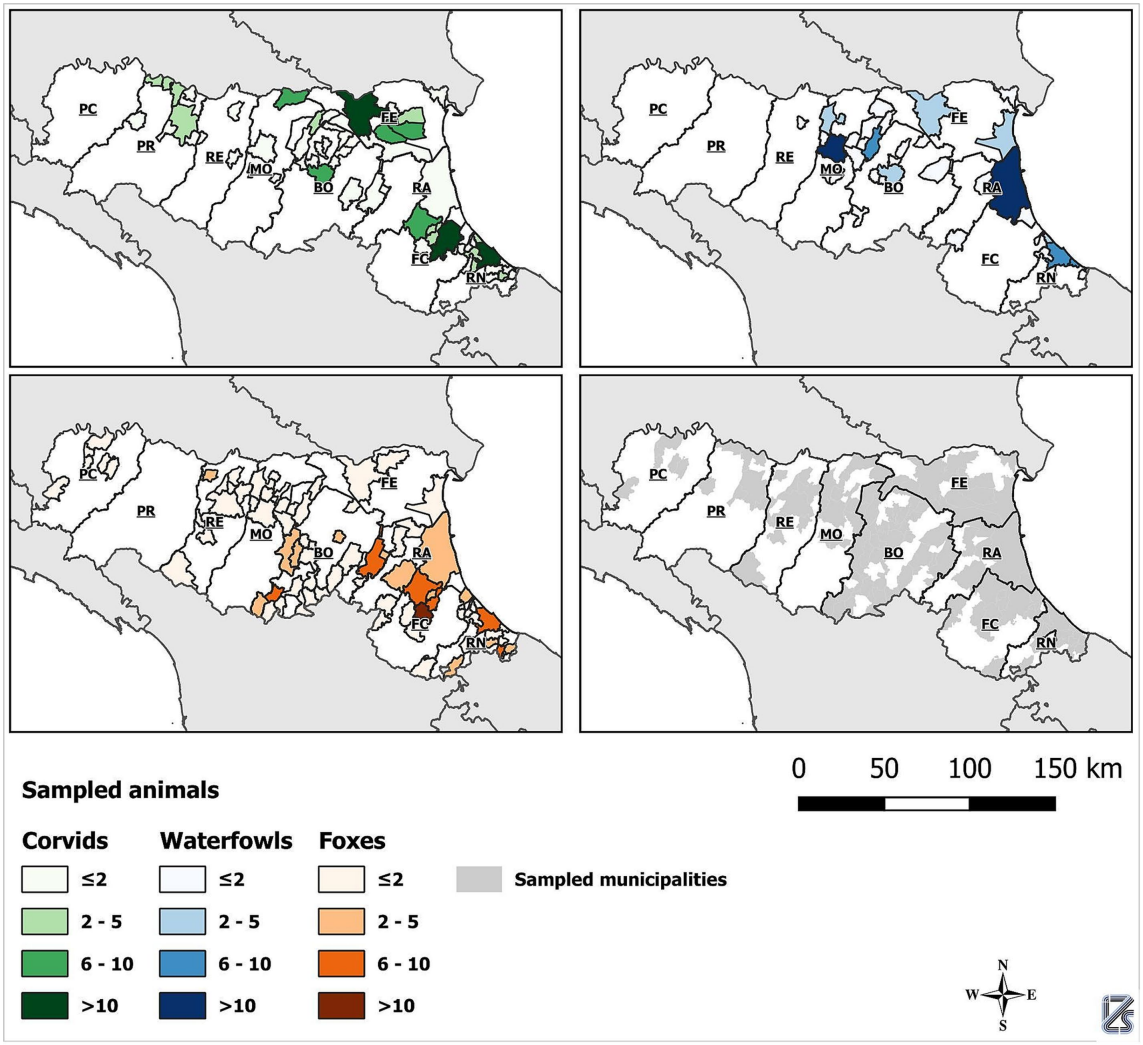
Distribution of *Klebsiella* spp. withing animal groups.

*Klebsiella* spp. were isolated from 32 out of 493 (6.53%) samples, namely 5 *K. aerogenes* (5/32; 15.6%), 10 *K. oxytoca* (31.3%), 10 *K. pneumoniae* (31.3%), 1 *K. quasipneumoniae* (3.1%) and 6 *K.* *variicola* (18.8%). Figure 2 shows the distribution of *Klebsiella* spp. according to the group of wild animals from which they were isolated. *K. aerogenes* showed an equal distribution between corvids (40%) and foxes (40%), with a lower prevalence in waterfowl (20%). *K. oxytoca* was isolated from corvids (50%) and foxes (50%). *K. pneumoniae* was primarily isolated from waterfowl (80%) followed by foxes (20%). The only strain of *K. quasipneumoniae* was found in corvids (100%). *K.* *variicola* was predominantly found in waterfowl (50%), with lower prevalence in corvids (33.3%) and foxes (16.7%).

Statistical testing confirmed that the distribution of *Klebsiella* spp. varied significantly across the three animal groups sampled. In particular, the analysis revealed a statistically significant association between animal group and *Klebsiella* species distribution (*χ*^2^ = 17.867, df = 8, *p* = 0.022). The Likelihood Ratio test further confirmed this finding (*χ*^2^ = 23.509, *p* = 0.003), suggesting that certain *Klebsiella* spp. were preferentially isolated from specific animal groups. Notably, *K. pneumoniae* showed a strong association with waterfowl (80.0%), while *K. oxytoca* was equally distributed between corvids and foxes (50.0% each).

### 3.2 Antimicrobial susceptibility of *Klebsiella* spp.

**Figure 3:**
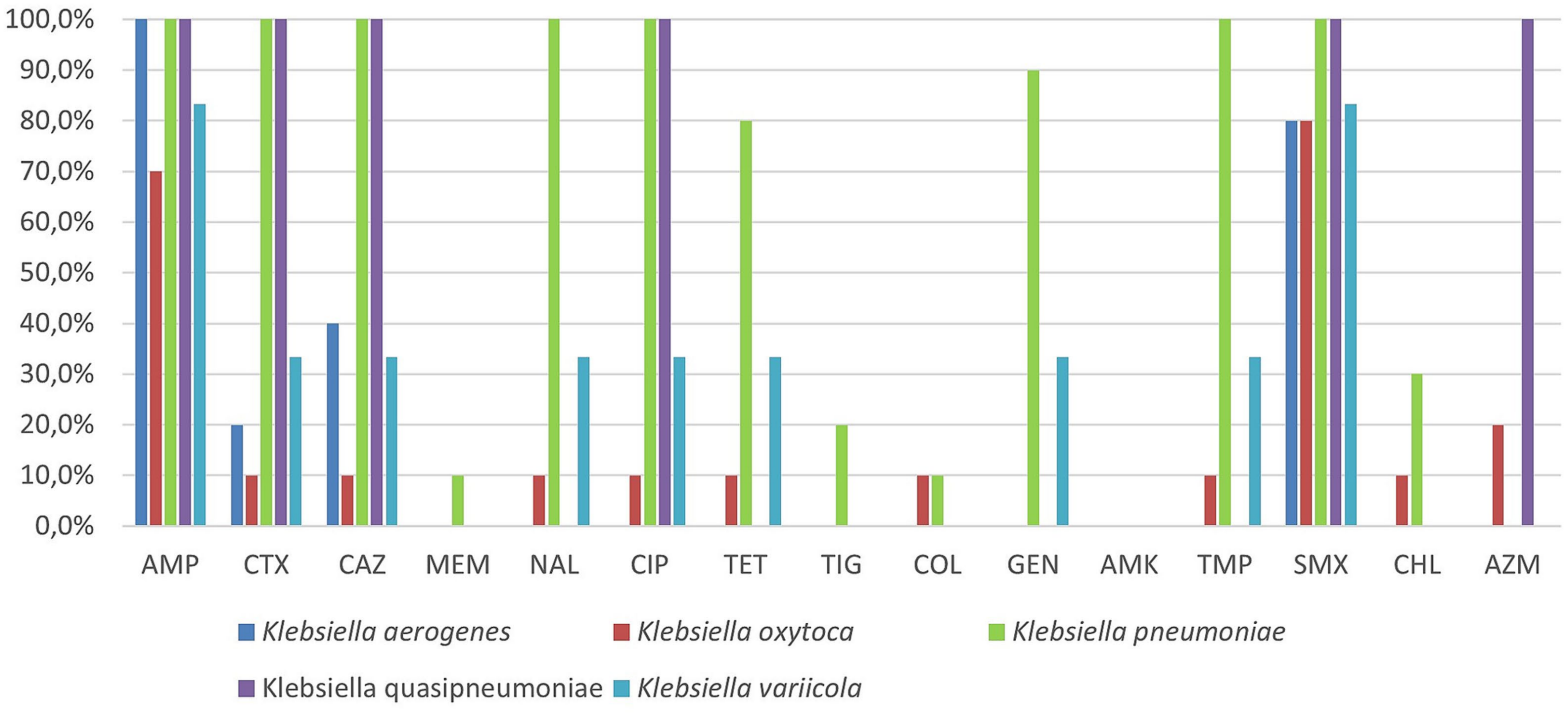
Proportion of AMR in *Klebsiella* spp. strains of wildlife origin (AMP, ampicillin; CTX, cefotaxime; CAZ, ceftazidime; MEM, meropenem; NAL, nalidixic acid; CIP, ciprofloxacin; TET, tetracycline; TIG, tigecycline; COL, colistin; GEN, gentamicin; AMK, amikacin; TMP, trimethoprim; SMX, sulfamethoxazole; CHL, chloramphenicol; AZM, azithromycin).

Figure 3 shows the resistance rates among the *Klebsiella* spp. isolates. As expected, 100% of *K. pneumoniae* isolates were intrinsically resistant to AMP (10/10; 95% CI: 69.2–100%), as well as 100% of *K. aerogenes* and *K. quasipneumoniae* strains, while resistance to AMP was detected in 3/10 (30%; 95% CI: 6.7–65.2%) *K. oxytoca* and 1/6 (16.7%; 95% CI: 0.4–64.1%) *K.* *variicola* isolates. Resistance to SMX was widespread, with a positivity rate of 100% in *K. pneumoniae* and *K. quasipneumoniae* isolates. Resistance to CTX, CAZ, and CIP was also common, as in 100% of *K. pneumoniae* isolates.

Resistance to GEN (34.4, 95% CI: 20.0–52.0%) and TET (34.4, 95% CI: 20.0–52.0%) was moderately widespread, with *K. pneumoniae* being the main contributor. MEM, COL, and TIG showed relatively low resistant rates across all species, with positivity rates of 3.1% (95% CI: 0.6–15.7%), 6.3% (95% CI: 1.7–20.4%), 6.3% (95% CI: 1.7–20.4%) respectively. No resistance to AMK (0%) was found across all species.

**Figure 4:**
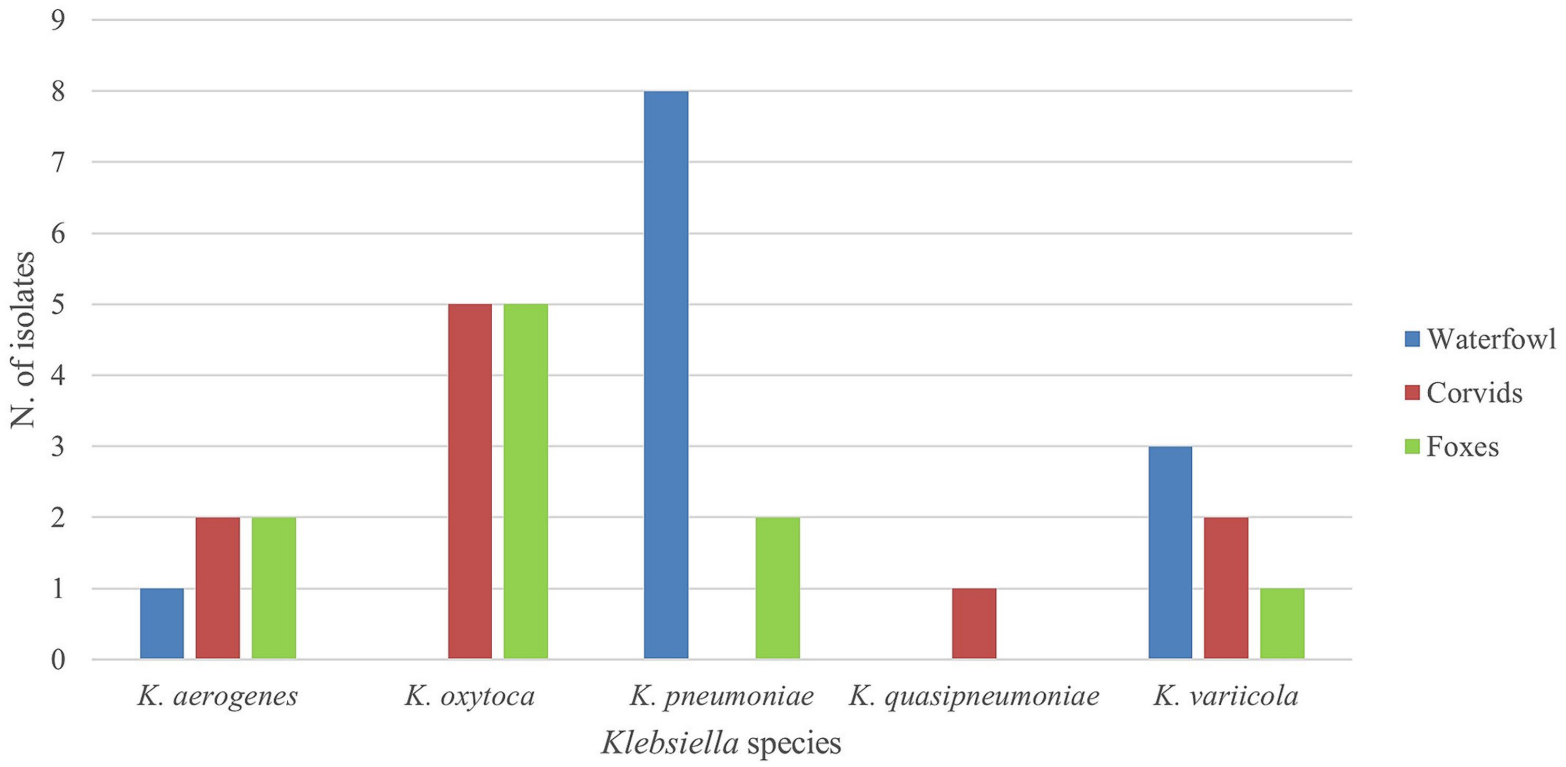
Distribution of MDR strains among *Klebsiella* spp. isolates.

MDR was observed in 14/32 (43.8, 95% CI: 27.0–62.0%) *Klebsiella* spp. isolates. *K. pneumoniae* was the species with the highest rate of MDR (10/14; 71.4, 95% CI: 44.8–89.7%). Moreover, *K. pneumoniae* showed also the highest level of multiresistance since 9 out of 10 strains (90, 95% CI: 59.6–98.2%) were resistant to more than six classes of antibiotics (Figure 4). The distribution of MDR among *Klebsiella* spp. isolates from different animal groups showed great variations in the resistance patterns. Waterfowl and foxes harbored the highest number of MDR isolates, being 10/14 (71.4, 95% CI: 44.8–89.7%) and 3/14 (21.4, 95% CI: 7.0–47.6%), respectively, whereas corvids contributed only with 1/14 isolate (7.1, 95% CI: 0.4–32.8%). The waterfowl isolates exhibited a high-level of MDR, since 8/14 strains (57.1, 95% CI: 32.2–79.2%) were resistant to 5 classes of antimicrobials. Foxes showed a unique profile, with resistance patterns skewed toward extreme levels (6–7 classes).

The analysis of MDR *K. pneumoniae* isolates revealed differences across animal groups. The 10 MDR isolates showed resistance to a minimum of 4 classes to a maximum of 6 classes of antibiotics. Waterfowl emerged as the primary reservoir of MDR *K. pneumoniae* isolates, harboring 8 out of 10 MDR *K. pneumoniae* (80.0, 95% CI: 51.9–94.7%). In addition, although only a few *K. pneumoniae* isolates were obtained from foxes (2/10, 20.0, 95% CI: 5.3–48.1%), all of them were MDR (2/2; 100%; 95% CI: 34.2–100%) and showed resistance to 6 antibiotics.

As shown in Table 1, six different resistance patterns were found among the 10 *K. pneumoniae* isolates. The MAR index ranged from 0.53 to 0.73.

**Table 1:** Phenotypic AMR profiles and MAR index of MDR *K. pneumoniae* isolates.

No of isolates	Resistance type	Phenotypic resistance patterns	MAR index
1	MDR	Four classes (AMP), CTX, CAZ, NAL, CIP, GEN, TMP, SMX	0.53
5	MDR	Five classes (AMP), CTX, CAZ, NAL, CIP, TET, GEN, TMP, SMX	0.60
1	MDR	Five classes (AMP), CTX, CAZ, NAL, CIP, GEN, TMP, SMX, CHL	0.60
1	MDR	Six classes (AMP), CTX, CAZ, NAL, CIP, TET, COL, GEN, TMP, SMX	0.67
1	MDR	Six classes (AMP), CTX, CAZ, MEM, NAL, CIP, TET, TIG, TMP, SMX, CHL	0.73
1	MDR	Six classes (AMP), CTX, CAZ, NAL, CIP, TET, TIG, GEN, TMP, SMX, CHL	0.73

### 3.3 Genomic analysis of *Klebsiella pneumoniae* isolates and *in-silico* identification of antimicrobial resistance genes

*In-silico* Sequence Typing (ST) revealed that all *K. pneumoniae* isolates belonged to ST307 and all shared the K locus KL102, as well as the O locus O1/O2v2. Analysis of WGS data revealed the absence of any virulence locus reported in the Kleborate database, while the several AMR genes were identified among the 10 *K. pneumoniae* isolates (Table 2).

**Table 2:** Genes and plasmid replicons identified among *K. pneumoniae* isolates.

Strain	Host	Detected gene(s)	Detected plasmid replicons
77	Waterfowl	aac(3)-IIa.v1^;aac(6′)-Ib-cr.v2;strA.v1^;strB.v1qnrB1.v2^sul2tet(A).v1dfrA14.v2*OXA-1; TEM-1D.v1^CTX-M-15SHV-28^GyrA-83I; ParC-80IcatB3.v1?-70%	IncFII(K), IncFIB(K), Col440I, Col156
79	Waterfowl	aac(3)-IIa.v1^;aac(6′)-Ib-cr.v2;strA.v1^;strB.v1qnrB1.v2^sul2tet(A).v1dfrA14.v2*OXA-1; TEM-1D.v1^CTX-M-15SHV-28^GyrA-83I; ParC-80IcatB3.v1?-70%	IncFII(K), IncFIB(K), Col440I, Col156
81	Waterfowl	aac(3)-IIa.v1^;aac(6′)-Ib-cr.v2;strA.v1^;strB.v1qnrB1.v2^sul2tet(A).v1dfrA14.v2*OXA-1; TEM-1D.v1^CTX-M-15SHV-28^GyrA-83I; ParC-80IcatB3.v1?-70%	IncFIB(K)
84	Waterfowl	aac(3)-IIa.v1^;aac(6′)-Ib-cr.v2;strA.v1^;strB.v1qnrB1.v2^sul2tet(A).v1dfrA14.v2*OXA-1; TEM-1D.v1^CTX-M-15SHV-28^GyrA-83I; ParC-80IcatB3.v1?-70%	IncFIB(K)
86	Waterfowl	aac(3)-IIa.v1^;aac(6′)-Ib-cr.v2;strA.v1^;strB.v1qnrB1.v2^sul2tet(A).v1dfrA14.v2*OXA-1; TEM-1D.v1^CTX-M-15SHV-28^GyrA-83I; ParC-80IcatB3.v1?-70%	IncFII(K), IncFIB(K), Col440I, Col156
89	Waterfowl	aac(3)-IIa.v1^;aac(6′)-Ib-cr.v2;strA.v1^;strB.v1qnrB1.v2^sul2tet(A).v1dfrA14.v2*OXA-1; TEM-1D.v1^CTX-M-15SHV-28^GyrA-83I; ParC-80IcatB3.v1?-70%	IncFII(K), IncFIB(K), Col440I, Col156
93	Fox	aac(6′)-Ib-cr.v2;strA.v1^;strB.v1qnrB1.v2^sul2tet(A).v1dfrA14.v2*OXA-1; TEM-1D.v1^CTX-M-15NDM-5SHV-28^GyrA-83I; ParC-80IcatB3.v1?-70%	IncX3, IncFII(K), IncFIB(K)
94	Fox	strA.v1^;strB.v1qnrB1.v2^sul2dfrA14.v2*TEM-1D.v1^CTX-M-15SHV-28^GyrA-83I; ParC-80I	IncFII(K), IncFIB(K)
180	Waterfowl	aac(3)-IIa.v1^;aac(6′)-Ib-cr.v2;strA.v1^;strB.v1qnrB1.v2^catA1^sul2dfrA14.v2*OXA-1; TEM-1D.v1^CTX-M-15SHV-28^GyrA-83I; ParC-80IcatB3.v1?-70%	IncFIB(K), IncI1-I(Alpha)
183	Waterfowl	aac(3)-IIa.v1^;aac(6′)-Ib-cr.v2;strA.v1^;strB.v1qnrB1.v2^sul2dfrA14.v2*OXA-1; TEM-1D.v1^CTX-M-15SHV-28^GyrA-83I; ParC-80IcatB3.v1?-70%	IncFIB(K)

^inexact nucleotide but exact amino acid match; *inexact nucleotide and inexact amino acid match; ?incomplete match.

All *K. pneumoniae* isolates carried at least one *β*-lactamase gene. ESBL genes were found in 10/10 (100.0, 95% CI: 72.2–100%) isolates, with the most frequent being *bla*_CTX-M-15_. All *bla*_CTX-M-15_-positive strains also harbored the *bla*_TEM-1D_ and *bla*_SHV-28_ genes. The carbapenemase *bla*_NDM-5_ gene was detected only in the strain No 93 (1/10; 10.0, 95% CI: 1.8–40.4%). The chromosomal *bla*_SHV-1_ was found in two isolates (2/10; 20.0%; 95% CI: 5.7–51.0%), whereas *bla*_SHV-28_ was the most frequently detected (10/10). Among the aminoglycoside resistance genes, the *aac(3)-IIa, aac(6′)-Ib-cr*, *strA*, and *strB* were present in all isolates (10/10). All *K. pneumoniae* isolates harbored fluoroquinolone resistance determinants, i.e., the gene *qnrB1* and the combination of chromosomal mutations in *gyrA* (GyrA-83I) and *parC* (ParC-80I) (10/10). The sulphonamide resistance gene *sul2* and the trimethoprim resistance gene *dfrA14* were found in all isolates. The *tetA* gene was detected in 7/10 (70.0, 95% CI: 39.7–89.2%) *K. pneumoniae* isolates. A gene with 70% identity to *catB3* was detected in 9/10 (90.0, 95% CI: 59.6–98.2%) *K. pneumoniae* isolates (Table 2).

With the aim of understanding the possible localization of the AMR genes and since it is well known that plasmids contribute to the spread of relevant resistance determinants, we performed a plasmid analysis on our collection of *K. pneumoniae* sequenced isolates. In particular, replicon types were defined and the presence of Mobile Genetic Elements (MGEs) associated with AMR were investigated in plasmids.

Replicon types analysis, on short-read assemblies, showed the presence of IncFIB(K) and IncFII(K) replicons among the *K. pneumoniae* isolates from wild birds and red foxes except the strain No 93 (Table 2). Additional colicin (Col)-type plasmids [e.g., Col440I, Col156, Col440I, Col(pHAD28), Col(IMGS31) were found in different hosts (Table 2)]. One strain from a fox (strain No 93 in Table 2), carrying the NDM-5 carbapenemase gene (*bla*_NDM-5_), exhibited an IncX3 replicon type, notoriously associated to plasmids with *bla*_NDM-5_ and in general with other *bla* AMR genes (Guo et al., 2022; Zhu et al., 2024).

**Figure 5:**
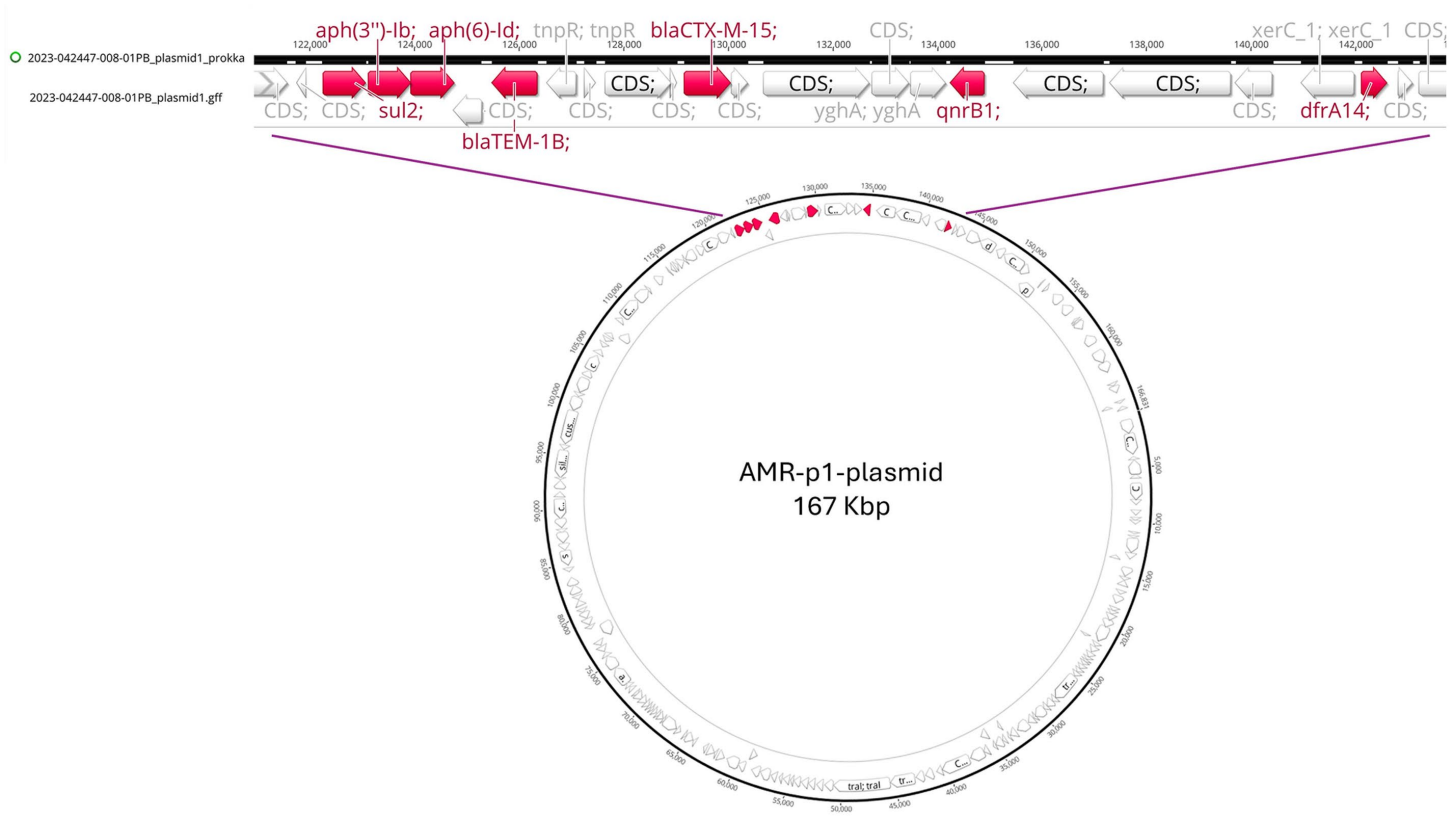
AMR-p1-plasmid map.

Moreover, 27 closed plasmids were assembled from the *K. pneumoniae* genomes and only one of them, here named “AMR-p1-plasmid,” harbored AMR genes. The “AMR-p1-plasmid” was a 167 Kbp long plasmid, extracted from the complete genome sequences of the strain No 94, with a replicon type classified as IncFII(K)/IncFIB(K). AMR-p1-plasmid carried 7 AMR genes (Figure 5) namely *bla*_TEM-1B_ and *bla*_CTX-M-15_ (b-lactams), *sul2* (sulfamethoxazole), *aph(6)-Id* and *aph(3″)-Ib* (streptomycin), *dfrA14* (trimethoprim), *qnrB1* (ciprofloxacin). Since these resistance genes were shared by 10 *K. pneumoniae* strains, we performed a comparative analysis (BWA-MEM mapping of the reads of each isolate against the sequence of AMR-p1-plasmid) to better understand if any other *K. pneumoniae* isolates harbored the same plasmid. The analysis revealed that the whole sequence of AMR-p1-plasmid detected in the strain No 94 was present in 9 *K. pneumoniae* strains (77, 79, 86, 89, 93, 180, 183, 81, 84). The strain LP-270 possessed a conserved proportion of AMR-p1-plasmid (78%) thus not including the 7 AMR genes mentioned above. AMR-p1-plasmid was not detected in strain No 24, confirmed by the lack of IncFII(K)/IncFIB(K) replicon types. These results suggested that the *K. pneumoniae* isolates having *bla_TEM-1B_, bla_CTX-M-15_, sul2, aph(6)-I, aph(3″)-Ib* and *dfrA14* genes (Table 2) are those that probably harbored the AMR-p1 plasmid of replicon type IncFII(K)/IncFIB(K).

### 3.4 Concordance of genominc and phenotypic antimicrobial data in *Klebsiella pneumoniae* isolates

To assess the reliability of the genomic predictions, we compared genotypic resistance profiles (presence/absence of resistance genes) with phenotypic antimicrobial susceptibility results, examining concordance and discordance patterns across all isolates. Phenotypic resistance to 3GCs (CTX and CAZ) was observed in all isolates harboring the ESBL genes (10/10, 100.0, 95% CI: 72.2–100%). The only carbapenem-resistant isolate (strain No 93, isolated from a fox) carried the NDM-5 carbapenemase gene (*bla*_NDM-5_), consistent with its meropenem resistance. All other isolates lacking carbapenemase genes were susceptible to meropenem. A total of 9/10 *K. pneumoniae* isolates (90.0, 95% CI: 59.6–98.2%) showed resistance to gentamicin based on phenotypic testing. All gentamicin-resistant isolates harbored the *aac(3)-IIa* gene. The *aac(6′)-Ib-cr* gene was found in most isolates (9/10; 90.0%), including one that was phenotypically susceptible to gentamicin. None of the isolates were resistant to amikacin, regardless of the presence of *aac(6′)-Ib-cr*, as observed in other studies (Bado et al., 2016; Eftekhar and Seyedpour, 2015). The fox isolate No 94 was phenotypically resistant to gentamicin but lacked *aac(3)-IIa*, *aac(6′)-Ib-cr*, and other known aminoglycoside resistance genes. The *strA* and *strB* genes (conferring resistance to streptomycin–not tested in this study -) were detected in all isolates. All ciprofloxacin- and nalidixic acid-resistant isolates (10/10; 100.0%) possessed the plasmid-mediated gene *qnrB1* and the chromosomal mutations in GyrA-83I and ParC-80I. Resistance to sulfamethoxazole and trimethoprim was concordant with the presence of *sul2* and *dfrA14* genes (10/10 isolates for both) respectively. Phenotypic tetracycline resistance was observed in all the isolates that carried the *tetA* gene. One isolate (No 94) was phenotypically resistant both to tetracycline and to tigecycline without carrying the encoding genes, thus suggesting potential differences in expression or additional resistance mechanisms affecting those antimicrobials. Another strain (No 93) carrying the *tetA* gene was phenotypically resistant both to tetracycline and tigecycline. The *catB3* gene fragment detected in 9 isolates showed poor correlation with phenotypic resistance, as most of these isolates were susceptible to chloramphenicol, indicating likely a truncation in *catB3* gene that abolished its function. Strain No 94 (isolated from a fox) lacked the gene, but it was phenotypically resistant.

## 4 Discussion

The assessment of AMR dissemination in wildlife in the Italian Emilia-Romagna Region was carried out considering the genus *Klebsiella*, as a representative of clinical concern of the Enterobacterales order. The bacteria belonging to this genus can be found in the microbiota of animals and humans, but also in soil, water, and other environmental sources (Wall et al., 2023). *Klebsiella* spp. are capable of acquiring various resistance mechanisms through vertical or horizontal gene transfer, making it crucial to study how they adapt to different conditions, including wildlife environments (Quintelas et al., 2024). This study provides a comprehensive epidemiological assessment of *Klebsiella* spp. in Italian wildlife, with prevalence, resistance patterns, and phenotypic characterization based on the complete dataset. Genomic analysis was then performed on *K. pneumoniae* isolates to provide mechanistic insights into the resistance phenotypes and lineages detected within this genus.

The detection of *K. pneumoniae* and other *Klebsiella* spp. in wild animals in Northern Italy highlights the role of wildlife as environmental sentinels for AMR. Although the overall prevalence of *K. pneumoniae* was relatively low (2.0, 95% CI: 1.0–3.7%), its isolation from multiple species–particularly waterfowl–confirms the environmental circulation of clinically relevant AMR bacteria in ecosystems not directly exposed to antibiotic pressure, in line with global epidemiological data on dissemination of ESBL-producing *K. pneumoniae* (Huang et al., 2025). Moreover, the isolation of a carbapenemase-producing strain (carrying the *bla*_NDM-5_ gene) from a fox is of concern, highlighting the introduction of genes for resistance to CIA into wildlife reservoirs (Mo et al., 2018).

When compared to the latest European Centre for Disease Prevention and Control (ECDC) surveillance data (ECDC, 2024), the *K. pneumoniae* isolates of this study exhibit higher resistance rates to nearly all antibiotic classes. Resistance to 3GCs (100.0%) and fluoroquinolones (100.0%) was particularly high, well above national (19.6 and 17.4%, respectively) and EU (9.3 and 8.8%, respectively) rates for human clinical isolates. Similarly, resistance to GEN reached 90.0%, compared to 11.6% in Italy and 6% in the EU. Only resistance to carbapenems showed comparable levels (10.0% vs. 9.3% in Italy). It is important to note that our data were obtained from isolates recovered using selective media, which may enrich for resistant strains and thus represent a non-random subpopulation of the total bacterial community. Our prevalence estimates should be considered as a minimum indication of resistance in this population, and non-selective sampling approaches would be needed to assess the true ecological distribution of susceptible versus resistant isolates. Moreover, the ECDC data refer exclusively to resistance phenotypes in *K. pneumoniae* responsible for human bloodstream infections, whereas our data were obtained from wild animals, i.e., from a context entirely removed from direct clinical exposure. This makes the observed resistance levels even more concerning, as they suggest a likely role of wildlife not only as sentinel of AMR, but as true amplifiers, collecting AMR bacteria from different environments (sewage treatment plants, rivers, etc.) despite their lack of direct antimicrobials exposure (Allen et al., 2010; Guenther et al., 2012; Bonardi and Pitino, 2019; Plaza-Rodríguez et al., 2021). The Multiple Antibiotic Resistance (MAR) indices observed (up to 0.73 in *K. pneumoniae* isolates) further support the hypothesis of high environmental exposure to anthropogenic AMR sources.

The phenotypic resistance profiles revealed the presence of resistant and MDR strains, especially among *K. pneumoniae* isolates from waterfowl and foxes, consistent with previous findings in wildlife (Bonnedahl et al., 2014; Chiaverini et al., 2022). These strains showed resistance to up to six antibiotic classes, including 3GCs, fluoroquinolones, and sulphonamides, which are classified as CIA for human medicine (World Health Organization, 2024). *K. pneumoniae*, *K* *variicola*, *K. quasivariicola*, and *K. aerogenes* are intrinsically resistant to ampicillin through production of the class A *β*-lactamase enzyme SHV in *K. pneumoniae*, or its orthologues LEN in *K. variicola* and *K. quasivariicola* (Wyres et al., 2020; Dong et al., 2022; Intra et al., 2023). Otherwise, *K. oxytoca* carries an intrinsic *bla*_OXY_ gene encoding the chromosomal class A β-lactamase OXY, which is typically produced at a low level to confer variable resistance to penicillins. In our study, 100% of the *K. pneumoniae*, *K. quasipneumoniae* and *K. aerogenes* were resistant to AMP, while only 70% of *K. oxytoca* and 83.3% of *K. variicola* isolates were resistant despite the presence of the β-lactamase producing genes. The AMP-susceptible phenotype likely resulted from reduced expression or functional variants of the chromosomal β-lactamase genes, as these enzymes (especially in *K. oxytoca*) are typically expressed at levels sufficient to confer resistance to penicillins. In such cases, lower expression or loss-of-function variants may lead to insufficient β-lactamase activity, resulting in a susceptible phenotype (Potter et al., 2018; Yang et al., 2022).

The predominance of ST307 among *K. pneumoniae* isolates underlines its role in the dissemination of AMR in the wildlife populations. ST307 is recognized as a high-risk clone associated with extended-spectrum β-lactamase (ESBL) production and carbapenem resistance in clinical settings (Heiden et al., 2020; Peirano et al., 2020; Cejas et al., 2022; González-Espinosa et al., 2024; Zhu et al., 2024). The presence of this clone in wild animals confirms its potential clinical origin, highlighting the role of wildlife as reservoirs and sentinels for AMR (Quintelas et al., 2024).

The phenotypic resistance patterns were largely confirmed by the genomic analysis. The presence of *bla*_CTX-M-15_, *bla*_TEM-1D_, and *bla*_SHV-28_ genes in ESBL-producing isolates was strongly correlated with phenotypic resistance to 3GCs, as well as ciprofloxacin resistance was confirmed by the presence of *qnrB1* together with the chromosomal mutations in *gyrA* (GyrA-83I) and *parC* (ParC-80I). Similarly, the trimethoprim- and sulfamethoxazole- resistance corresponded with the presence of *dfrA14* and *sul2* genes, respectively.

The aminoglycoside resistance genes *aac(3)-IIa* and *aac(6′)-Ib-cr*, detected in most gentamicin-resistant isolates, confirm their role in aminoglycoside inactivation and it was confirmed by the phenotypic resistance profile of the strains (Ramirez and Tolmasky, 2010). A notable exception was observed in a strain isolated from a fox (No 94), that showed phenotypic resistance to gentamicin and chloramphenicol despite lacking known resistance genes (*aac(3)-IIa*, *catB3*, etc.). This anomaly suggests the presence of alternative mechanisms, such as chromosomal mutations, efflux pump overexpression, or resistance genes not currently collected in databases (Li et al., 2023; Padilla et al., 2010; Tsai et al., 2017). Interestingly, no isolates were resistant to amikacin, despite the presence of the *aac(6′)-Ib-cr* determinant, confirming the limited contribution of this gene without co-factors to clinically relevant resistance (Ramirez and Tolmasky, 2010). Concerning tetracycline, the presence of the *tetA* gene was always associated with resistance to tetracycline but did not confer resistance to tigecycline, in agreement with previous studies highlighting tigecycline’s efficacy against many tetracycline-resistant *K. pneumoniae* strains (Sader et al., 2025). Also, in this case, the strain No 94 represented an exception, as it was phenotypically resistant to both tetracycline and tigecycline despite lacking the *tetA* gene. Finally, sulphonamide and trimethoprim resistance genes (*sul2* and *dfrA14*, respectively) were distributed in all resistant isolates, demonstrating their persistence in bacteria of wildlife origin (Wang et al., 2023).

Overall, the high concordance between genotypic and phenotypic resistance data (>90% for most antimicrobials) validates the utility of genomic analysis for predicting resistance phenotypes in *K. pneumoniae*. However, the discordant cases identified (particularly isolate No 94 with gentamicin and tetracycline resistance without corresponding known genes) suggest the presence of additional or alternative resistance mechanisms not identified by current sequence-based prediction tools. These findings underscore the importance of integrating both phenotypic and genomic approaches in comprehensive AMR characterization studies.

The analysis of replicons revealed the presence of IncF-type replicons (IncFII(K), IncFIB(K)) in the majority of the isolates, probably associated to the presence of the AMR-p1 plasmid, which harbored the AMR genes detected in these isolates (i.e. *bla*_TEM-1B_*, bla*_CTX-M-15_*, sul2, aph(6)-I, aph(3″)-Ib and dfrA14*). These replicons are known to harbor multiple resistance genes and play a crucial role in their horizontal transfer (Carattoli, 2009; Bi et al., 2018; Rozwandowicz et al., 2018; Oliveira et al., 2020; Abdelwahab et al., 2021; Wang et al., 2021). The identification of IncX3 replicon in a fox isolate carrying also *bla*_NDM-5_ (strain No 94) suggested the possible involvement of IncX3 plasmid in the spread of the *bla*_NDM-5_ gene within a One Health context and underline the potential zoonotic risk associated with wildlife, as this replicon is commonly implicated in the spread of carbapenemases in clinical settings (Liakopoulos et al., 2016; Shao et al., 2020; Takei et al., 2022; Zeng et al., 2022; Menezes et al., 2023).

The presence of the colicinogenic (Col) plasmid type was identified in some isolates, including Col440I, Col156, Col(pHAD28), and Col(IMGS31). Usually, those plasmids are associated with bacteriocin production, they are generally not considered primary vectors of multidrug resistance, but can play a role in interbacterial competition, particularly in complex ecological niches such as the gut or environmental reservoirs (Rozwandowicz et al., 2018; Han et al., 2025). Moreover, they are frequently detected in pathogenic strains and hospital settings all over the world (Shelenkov et al., 2023; Amadesi et al., 2024; Loconsole et al., 2024; AL Shizawi et al., 2025). In the present study, Col-type replicons were more frequently detected in strains isolated from waterfowl and corvids, while they were absent in fox isolates. Their presence may reflect environmental selective pressures supporting bacterial competitiveness rather than AMR resistance (Carattoli, 2009; Janecko et al., 2018; Shankar et al., 2020; Moser et al., 2022).

Considering the ecological origin of the isolates, differences in AMR profiles were apparent. The isolates from waterfowl showed a high prevalence of ESBL genes and aminoglycoside-resistance markers, reflecting a potential exposure to environmental contamination from human sources, such as wastewater or agricultural discharge (Allen et al., 2010). Of particular concern was the strain of fox origin with unusual carbapenem-resistance and harboring multiple resistance genes. Red foxes are widespread opportunistic predators, capable of adapting their diet to seasonal and local availability, including fruits, invertebrates, small mammals, birds, and even human food waste. Their broad ecological plasticity and frequent proximity to urban areas make them one of the best sentinel species for AMR surveillance (Mo et al., 2018; Garcês and Pires, 2021). These traits likely contribute to their exposure to resistant bacteria both through environmental routes (e.g., sewage, garbage, wastewater) and the consumption of contaminated preys. Such findings support the hypothesis that red foxes may act as reservoirs and vectors facilitating the environmental dissemination of multi-resistant *K. pneumoniae* (Guenther et al., 2012).

## 5 Conclusion

This study provides the evidence of ESBL and carbapenemase-producing *Klebsiella* spp. in wild birds and red foxes in Northern Italy. Our interest was particularly focused on the *K. pneumoniae* isolates, due to their clinical importance in human health. The high prevalence of MDR *K. pneumoniae* strains, the presence of clinically relevant resistance genes such as *bla*_CTX-M-15_ and *bla*_NDM-5_, and the detection of plasmids carrying AMR genes emphasize the importance of wildlife in the spread of AMR through different geographical areas. The results of this study confirm that wildlife can be effectively used to monitor the environmental spread of AMR, especially for red foxes and waterfowl, that can serve as valuable sentinels. Due to their differing feeding behaviors, ecological niches and degrees of synanthropy, these animals could provide complementary information: foxes may reflect short-range AMR dissemination within terrestrial ecosystems, while waterfowl–given their mobility and migratory patterns–can serve as indicators of long-range environmental AMR dispersion.

Furthermore, the high rates of AMR *K. pneumoniae* of wildlife origin, compared to those reported for clinical human isolates in the EU, suggest the likely role of wild animals as capable of amplifying AMR along the human/environmental/animal interface. Although this study was not designed to establish direct transmission links between wildlife and human populations, the presence of MDR and high-risk *K. pneumoniae* clones in natural ecosystems provides indirect but significant evidence of AMR environmental circulation. From a One Health perspective, the detection of such clinically relevant strains in wildlife highlights the need for integrated surveillance systems that recognize wildlife as a strategic component of broader antimicrobial resistance monitoring and prevention strategies.

## Data Availability

The datasets presented in this study can be found in online repositories. The names of the repository/repositories and accession number(s) can be found at: https://www.ebi.ac.uk/ena/browser/view/PRJEB77858.

## Footnotes

1
https://bigsdb.pasteur.fr/klebsiella/klebsiella.html
